# Morbidity and mortality related to pneumonia and TRACHEOBRONCHITIS in ICU after lung transplantation

**DOI:** 10.1186/s12890-018-0605-9

**Published:** 2018-03-05

**Authors:** Sebastien Tanaka, Claire Geneve, Gianpiero Tebano, Nathalie Grall, Pascal Piednoir, Régis Bronchard, Mathieu Godement, Enora Atchade, Pascal Augustin, Herve Mal, Yves Castier, Philippe Montravers, Mathieu Desmard

**Affiliations:** 1 APHP, CHU Bichat-Claude Bernard, Département d’Anesthésie Réanimation, 46 rue Henri Huchard, 75018 Paris, France; 2 Université Denis Diderot, PRESS Sorbonne Cité, Paris, France; 3 INSERM, UMR 1137, Infection, Antimicrobiens, Modélisation, Evolution, Paris, France; 4 AP-HP, CHU Bichat-Claude Bernard Laboratoire de Microbiologie, Paris, France; 5 APHP, CHU Bichat-Claude Bernard, Service de Pneumologie B et Transplantation Pulmonaire, Paris, France; 6 INSERM, UMR1152. Physiopathologie et Epidémiologie des Maladies Respiratoires, Paris, France; 7 APHP, CHU Bichat-Claude Bernard, Service de Chirurgie Thoracique et Vasculaire, Paris, France; 8 Service de Réanimation, Centre Hospitalier Sud Francilien, Corbeil-Essonnes, France

**Keywords:** Lung transplantation, Pneumonia, Bronchitis, Mortality, ICU

## Abstract

**Background:**

Bacterial respiratory infections (BRI) are major complications contributing to increased morbidity and mortality after lung transplantation (LT). This study analyzed epidemiology and outcome of 175 consecutive patients developing BRI in ICU after LT between 2006 and 2012.

**Methods:**

Three situations were described: colonization determined in donors and recipients, pneumonia and tracheobronchitis during the first 28 postoperative days. Severity score, demographic, bacteriologic and outcome data were collected.

**Results:**

26% of donors and 31% of recipients were colonized. 92% of recipients developed BRI, including at least one episode of pneumonia in 19% of recipients. Only 21% of recipients developed BRI with an organism cultured from the donor’s samples, while 40% of recipients developed BRI with their own bacteria cultured before LT. Purulent sputum appears to be an important factor to discriminate tracheobronchitis from pneumonia. When compared to patients with tracheobronchitis, those with pneumonia had longer durations of mechanical ventilation (13 [3–27] vs 3 [29], *p* = 0.0005) and ICU stay (24 [16–34] vs 14 [9-22], *p* = 0.002). Pneumonia was associated with higher 28-day (11 (32%) vs 9 (7%), *p* = 0.0004) and one-year mortality rates (21 (61%) vs 24 (19%), *p* ≤ 0.0001).

**Conclusions:**

These data confirm the high frequency of BRI right from the early postoperative period and the poor prognosis of pneumonia after LT.

## Background

Among the many issues reported in the early postoperative phase after lung transplantation (LT), bacterial respiratory infections (BRI) constitute major complications largely contributing to increased mortality in transplant recipients [1–6]. The etiologies of these infections are very diverse, involving community bacteria and hospital-acquired micro-organisms [4–7]. In a large cohort of 170 patients, Riera et al. recently demonstrated, for the first time, an increased mortality rate of ventilator-associated pneumonia (VAP) following LT [1].

Although recent guidelines have defined pneumonia, tracheobronchitis and colonization after lung and heart transplantation [8], the distinctions between these entities are blurred by several confounding factors such as ischemia-reperfusion syndrome, immunosuppression or atelectasis [9]. Although a consensus has been reached concerning the treatment of bacterial pneumonia [8], the need to extend antibiotic therapy to tracheobronchitis remains controversial. Criteria usually proposed for the diagnosis of postoperative pneumonia in LT recipients are difficult to identify, resulting in a high risk of missing pneumonia in these immunocompromised patients [8]. Our local policy is to initiate antibiotic therapy as soon as a patient is suspected of having BRI, even in the absence of criteria of pneumonia.

The aim of the present study was to compare the characteristics of patients who developed one or more episodes of BRI during the first 28 days after LT in ICU with those who did not. The influence of colonization of the bronchial tree and tracheobronchitis on the development of pneumonia and the role of these entities on the outcome were also evaluated.

## Methods

### Study population

Between January 2006 and December 2012, all consecutive adult patients undergoing LT and admitted to the intensive care unit (ICU) for postoperative care for at least 24 h were retrospectively included in a database. The study period included the length of ICU stay during the first 28 days following transplantation. According to French law, no informed consent was required in view of the observational and retrospective nature of this study. The study was approved by the Paris North Hospitals Institutional Review Board (Paris VII University, AP-HP, IRB No. 00006477).

### Perioperative management

Surgical transplantation procedure is standardized according to our local policy [10, 11]. Perioperative care, including postoperative management, was standardized for all patients according to our standard protocol [12]. Immunosuppressive therapy was based on a combination of prednisolone, ciclosporin and azathioprine subsequently replaced by mycophenolate mofetil.

In patients without previous colonization of the respiratory tract, perioperative antibacterial prophylaxis consisted of cefamandole at induction of anesthesia and continued for 48 h. In patients with preoperative colonization, prophylaxis was adapted to the micro-organisms isolated. Our center’s policy does not include digestive or bronchial decontamination prior to LT.

### Microbiologic samples

Respiratory colonization samples were performed in a large proportion of patients more than 6 months before transplantation. Respiratory samples are not routinely performed immediately prior to transplantation in our center.

Pretransplant tracheal bacterial colonization was recorded. Donor airway samples were obtained by bronchial aspiration during the surgical procedure before transplantation.

During the postoperative period, fiberoptic bronchoscopy was performed daily with invasive respiratory tract samples collected by bronchial aspiration (BA) every two or three days and by bronchoalveolar lavage [13] in the case of suspected viral or fungal infection. Microbiologic samples were processed according to the usual techniques. Susceptibility testing was performed as previously described [14].

### Definitions of BRI

**Pneumonia** was defined as positive culture of BA samples yielding ≥10^5^ colony-forming units (CFU)/mL combined with new or persistent lung infiltrate on chest X-ray, and two or more of the following criteria: temperature ≥ 38.4 °C or < 36 °C, WBC count >11,000 /mm^3^ or <4000 /mm^3^, at least 30% decrease of PaO_2_/FiO_2_ ratio, and purulent secretions [15]. **Tracheobronchitis** was defined as positive culture of microbiologic samples, normal appearance or moderate interstitial infiltrates on chest X-ray and at least one of the previously described clinical signs [16]. **Colonization** was defined as positive culture of microbiologic samples with no clinical, laboratory or radiological signs [8].

### Antibiotic therapy

According to local policy, all cases of suspected postoperative BRI (pneumonia or tracheobronchitis) were treated by empiric antimicrobial therapy, followed, when the diagnosis was confirmed, by targeted therapy according to susceptibility testing for a total duration of seven days. Our local empiric antimicrobial therapy consisted of a combination of cefepime and amikacin.

### Data collection

Demographic characteristics, underlying diseases and clinical data were recorded. Severity scores (SOFA and SAPS II) were assessed on ICU admission. At the time of pulmonary samples, clinical characteristics (purulent secretions, abundance of secretions, temperature), laboratory parameters (white blood cell (WBC) count, PaO2), chest X-ray imaging and results of microbiologic cultures were collected. Morbidity criteria (ICU length of stay, duration of mechanical ventilation, primary graft dysfunction score) and 28-day and one-year mortality after LT were assessed [17].

### Statistical analysis

All statistical analyses were performed using GraphPad Prism 5.0 (Apple, Cupertino, CA). Quantitative variables are expressed as median and interquartiles (25th–75th percentiles) and were compared by a nonparametric Mann-Whitney test. Categorical variables are expressed as proportions and absolute numbers, and were compared by Chi-square test or Fisher’s exact test. In the subgroup of patients with BRI, we compared the clinical presentation, microbiologic data and outcome of patients with and without criteria of pneumonia. Risk factors for one-year mortality were analyzed by univariate analysis. Variables with a *p*-value< 0.15 on univariate analysis were entered in a multivariate logistic regression model. One-year mortality was estimated by the Kaplan-Meier method. A *p*-value < 0.05 was considered statistically significant.

## Results

### Demographic and clinical characteristics

Between 2006 and 2012, 175 LT were performed. Demographic data are presented in Table 1.

**Table 1:** Demographic and clinical data

Variable	Total population (*n* = 175)	Patients without BRI (*n* = 14)	Patients with BRI (*n* = 161)	*p	patients with pneumonia (*n* = 34)	Patients with tracheobronchitis without any episode of pneumonia (*n* = 127)	*p
Age, year, median [IQR]	57 [50–60]	60 [55–63]	57 [50–60]	0.016	56 [50–59]	57 [49–60]	0.84
Male gender, n (%)	118 (67)	6 (42)	112 (69)	0.07	24 (71)	88 (69)	1
Underlying lung disease, n (%)							
Emphysema/COPD	70 (40)	5 (36)	65 (40)	0.78	14 (41)	51 (40)	1
Idiopathic pulmonary fibrosis	66 (38)	7(50)	59 (37)	0.39	9 (27)	50 (39)	0.23
Other	39 (22)	2 (14)	37 (23)	0.74	11 (32)	26 (21)	0.17
Transplantation type, n (%)							
Single lung	125 (71)	13 (92)	112 (69)	0.07	20 (59)	92 (72)	0.4
Double	50 (29)	1 (8)	49 (31)	0.1	14 (41)	35 (28)	0.14
SAPS II, median [IQR]	35 [29–42]	32 [28–36]	35 [29–42]	0.34	37 [32–48]	35 [28–42]	0.09
SOFA	5 [3–8]	4 [3–6]	5 [3–8]	0.41	7 [4–10]	5 [3–7]	0.06
ECLS, n (%)	30 (17)	2 (14)	28 (17)	0.76	12 (35)	16 (13)	0.004
Primary graft dysfunction, n (%)	12 (7)	1 (7)	11 (7)	0.96	2 (6)	9 (7)	0.06
Donor colonization prior to LT, n (%)	47 (42)	3 (21)	44 (27)	0.76	9 (47)	35 (42)	0.8
One micro-organism	39 (83)	2 (67)	37 (84)	0.4	8 (89)	29 (83)	1
More than one micro-organism	8 (17)	1 (33	7 (16)	0.4	1 (11)	6 (17)	1
Recipient colonization prior to LT, n (%)	55 (38)	8 (61)	47 (36)	0.08	10 (34)	37 (36)	1
One micro-organism	35 (64)	6 (75)	29 (62)	0.7	6 (60)	23 (62)	1
More than one micro-organism	20 (36)	2 (25)	18 (38)	0.7	4 (40)	14 (38)	1

A *p < 0.05 was considered statistically significant. LT = lung transplantation; COPD = Chronic obstructive pulmonary disease; ECLS = extracorporeal lung support; MV = mechanical ventilation; SOFA = Sequential Organ Failure Assessment; SAPSII = Simplified Acute Physiology Score

One hundred sixty one (92%) patients required mechanical ventilation during their ICU stay, and 64% of patients were mechanically ventilated at the time of diagnosis of BRI. A median of 7 [5–13] BAs were performed per patient yielding 467 bacteria (Table 2**)**.

**Table 2:** Bacterial isolates cultured from pulmonary samples in the postoperative period

Bacteria	All bacteria (*n* = 467)	Bacterial species involved in pneumonia (*n* = 51)	Bacterial species involved in tracheobronchitis (*n* = 416)	*p
Gram-positive bacteria, n (%)	172 (37)	17 (34)	155 (37)	0.64
- Coagulase-negative *Staphylococci*	20 (4)	3 (6)	17 (4)	0.47
*- Staphylococcus aureus*	47 (10)	3 (6)	44 (10.5)	0.45
*- Streptococcus* spp	77 (17)	9 (18)	68 (16)	0.84
*- Enterococcus* spp	25 (5.5)	2 (4)	23 (5.5)	1
- Other Gram-positive bacteria	3 (0.5)	0 (0)	3 (1)	1
Gram-negative bacteria, n (%)	295 (63)	34 (66)	261 (63)	0.64
*Enterobacteriaceae*	167 (36)	17 (33)	150 (36)	0.75
*Enterobacter* spp	40 (8.5)	7 (13)	33 (8)	0.18
*Escherichia coli*	42 (9)	3 (6)	39 (9)	0.6
*Klebsiella* spp	24 (5)	2 (4)	22 (5)	1
*Serratia marcescens*	15 (3)	1 (2)	14 (3.5)	1
*Proteus* spp	12 (2.5)	1 (2)	11 (2.5)	1
*Hafnia alvei*	18 (4)	2 (4)	16 (4)	1
*Citrobacter* spp	8 (2)	0 (0)	8 (2)	1
*Morganella morganii*	8 (2)	1 (2)	7 (2)	0.6
*Haemophilus influenzae*	19 (4)	2 (4)	17 (4)	1
Non-fermenting bacilli	88 (18.5)	12 (23)	76 (19)	0.34
*Pseudomonas aeruginosa*	62 (13)	7 (13)	55 (14)	0.83
*Stenotrophomonas maltophilia*	16 (3)	4 (8)	12 (2.8)	0.08
*Acinetobacter baumannii*	8 (2)	0 (0)	8 (2)	1
Other Non-fermenting	2 (0.5)	1 (2)	1 (0.2)	0.2
Gram-negative bacilli				
- *Neisseria* spp	13 (2.5)	1 (2)	12 (2.8)	1
- *Branhamella catarrhalis*	2 (0.5)	1 (2)	1 (0.2)	0.2
- Other Gram-negative bacteria	6 (1.5)	1 (2)	5 (1)	0.5

Expressed in number (%).*p < 0.05 was considered statistically significant

During the first 28 days after LT, 161 (92%) patients developed at least one episode of BRI. A median of 2 [1–3] episodes of BRI were observed per patient for a total of 380 episodes. In this population, 127 (73%) patients developed tracheobronchitis with no episode of pneumonia and 34 (19%) patients developed one or more episodes of pneumonia. Fourteen (8%) patients did not experience any BRI.

### Bacterial colonization

Forty-seven donors and 55 recipients were colonized before transplantation. Bacterial species involved in colonization are presented in Table 3.

**Table 3:** Type of donor and recipient bronchial colonization prior to LT

Bacteria	Donor’s bacteria (*n* = 51)	Recipient’s bacteria (*n* = 76)	*p
Gram-positive bacteria, n (%)	31 (61)	16 (21)	< 0.0001
- Coagulase-negative *Staphylococci*	1 (2)	0 (0)	0.4
*- Staphylococcus aureus*	15 (29)	12 (16)	0.08
*- Streptococcus* spp	11 (22)	4 (5)	0.0096
- Other Gram-positive bacteria	4 (8)	0 (0)	0.024
Gram-negative bacteria, n (%)	20 (39)	60 (79)	< 0.001
- *Enterobacteriaceae*	12 (23)	22 (29)	0.54
*Enterobacter* spp	2 (4)	6 (8)	0.47
*Escherichia coli*	5 (9)	7 (9)	1
*Klebsiella* spp	0 (0)	5 (7)	0.08
*Serratia marcescens*	0 (0)	2 (2.5)	0.51
*Proteus* spp	2 (4)	1 (1.25)	0.56
*Hafnia alvei*	2 (4)	0 (0)	0.16
*Citrobacter* spp	1 (2)	1 (1.25)	1
- *Haemophilus influenzae*	3 (6)	9 (12)	0.36
- Non-fermenting Gram-negative bacilli	2 (4)	24 (31,75)	< 0.0001
*Pseudomonas aeruginosa*	1 (2)	20 (26,75)	0.002
*Stenotrophomonas maltophilia*	1 (2)	4 (5)	0.64
- *Neisseria* spp	0 (0)	1 (1.25)	1
- *Branhamella catarrhalis*	0 (0)	4 (5)	0.15
- Other Gram-negative bacteria	3 (6)	0 (0)	0.06

*p < 0.05 was considered statistically significant

**Fig. 1:**
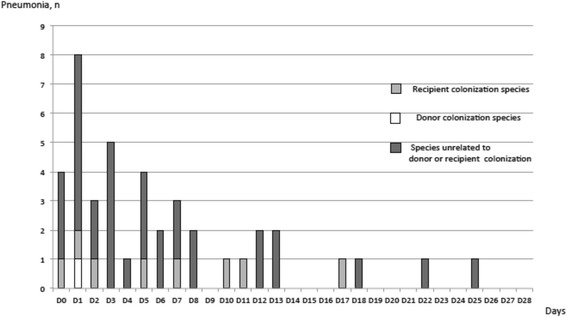
Relationship between pneumonia and recipient-donor colonization

Twenty-two colonized recipients developed postoperative BRI with one of their own micro-organisms cultured before LT (*Pseudomonas aeruginosa* (*n* = 9), *Staphylococcus aureus* (*n* = 6), *Streptococcus* spp. (*n* = 2), *Klebsiella* spp. (*n* = 2), *Enterobacter* spp. (*n* = 2), *Escherichia coli* (*n* = 2), *Stenotrophomonas* spp. (*n* = 2) and *Achromobacter* spp. (*n* = 1)). Twelve recipients developed BRI with a micro-organism cultured from the donor’s samples (*Staphylococcus aureus* (*n* = 6), *Streptococcus* spp. (*n* = 2), *Escherichia coli* (*n* = 1), *Pseudomonas aeruginosa* (*n* = 1), *Enterobacter* spp. (*n* = 1) and *Proteus* spp. (*n* = 1)). Data are presented in Fig. 1. Statistical analysis of bacterial colonization could not be performed due to missing data.

### Tracheobronchitis

Three hundred thirty-eight cases of tracheobronchitis were observed with a median time to onset of the first episode of 6 [1–12] days after LT.

### Pneumonia following LT

Overall, 42 episodes of pneumonia involving 51 micro-organisms were diagnosed in 34 patients with a median time to onset of 5 [1–8] days after LT (Table 1). No significant microbiologic differences were observed between the micro-organisms isolated from patients treated for pneumonia and tracheobronchitis (Table 2).

**Fig. 2:**
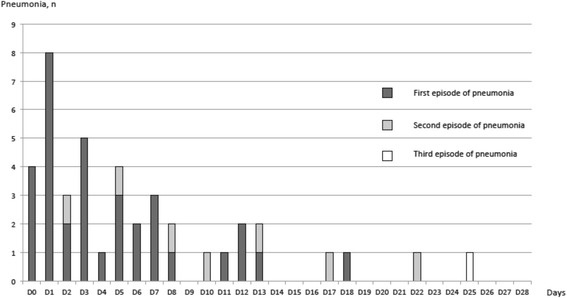
Time-course of episodes of pneumonia (*n* = 42)

Pneumonia occurred first in 17/175 patients (10%), while tracheobronchitis was reported before the onset of pneumonia in 158/175 (90%) patients. Time to onset of pneumonia is shown in Fig. 2**.**

Seven (17%) of the 42 episodes of pneumonia were caused by micro-organisms initially cultured from tracheobronchitis (*Klebsiella pneumoniae* (*n* = 1), *Enterobacter aerogenes* (*n* = 1), *Stenotrophomonas maltophilia* (*n* = 3), *Pseudomonas aeruginosa* (*n* = 1), *Enterococcus faecium* (*n* = 1), *Hafnia alvei* (*n* = 1) and *Achromobacter xyloxidans* (*n* = 1)).

No significant difference in the frequency of pneumonia due to organisms cultured from the host or the donor was observed between single and double lung transplantation (4/20 patients versus 5/14 patients, respectively, *p* = 0.43).

### Clinical characteristics of the patients in the various subgroups

Apart from age, no significant differences were observed between patients with and without BRI in terms of demographic or severity characteristics (Table 1**).**

No significant demographic differences were observed patients with pneumonia and patients with tracheobronchitis with no episode of pneumonia apart from the need for ECLS (Table 1**),** while they presented different clinical features at the time of pulmonary sampling (Table 4).

**Table 4:** Rates of clinical, radiologic and laboratory signs assessed at the time of pulmonary samples

	Pneumonia (*n* = 42)	Tracheobronchitis (*n* = 338)	*p
Fever or hypothermia, n (%)	12 (28)	42 (12)	0.009
Leukocytosis or leukopenia, n (%)	37 (88)	207 (71)	0.024
Purulent sputum, n (%)	34 (89)	72 (25)	< 0.0001
PaO_2_/FiO_2_ ratio < 240, n (%)	21 (63)	104 (59)	0.7
Chest X-ray infiltrates, n (%)	42 (100)	252 (74)	0.0001
Normal chest X-ray, n (%)	0 (0)	60 (19)	0.0003
Mechanical ventilation, n (%)	33 (79)	185 (55)	0.003

A *p < 0.05 was considered statistically significant

### Outcome

**Fig. 3:**
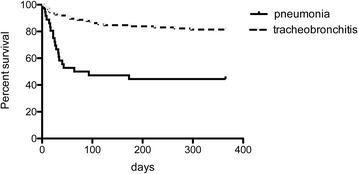
Kaplan-Meier graph of 1-year survival of patients with tracheobronchitis and pneumonia (*p*<0.0001 by log rank test)

Patients with at least one episode of pneumonia had higher 28-day and one-year mortality rates (Fig. 3) and longer duration of mechanical ventilation and ICU stay than patients with tracheobronchitis without pneumonia (Table 5**)**.

**Table 5:** Outcome data (LOS in ICU, duration of MV and mortality)

	Total population (*n* = 175)	Patients without BRI (*n* = 14)	Patients with BRI (*n* = 161)	*p	Patients with pneumonia (*n* = 34)	Patients with tracheobronchitis with no episode of pneumonia (*n* = 127)	*p
LOS in ICU (days)	15 [9–25]	8 [5–12]	15 [9–25]	0.0002	24 [16–34]	14 [9–22]	0.002
Duration of MV (days)	3 [2–14]	2 [1–3]	3 [2–14]	0.002	13 [3–27]	3 [2–9]	0.0005
28-day mortality, n (%)	23 (13)	3 (21)	20 (12)	0.4	11 (32)	9 (7)	0.0004
1-year mortality, n (%)	51 (29)	6 (42)	45 (27)	0.2	21 (61)	24 (19)	< 0.0001

LOS = length of stay; MV = mechanical ventilation. A *p < 0.05 was considered statistically significant

Univariate and multivariate analyses identified 3 independent factors of one-year mortality (Table 6). Pneumonia (OR 7.1 [2.7–18.7], *p* < 0.0001); age > 60 years (OR 3.0 [1.3–7.3], *p* = 0.013), and mechanical ventilation > 14 days (OR 5.1 [2.2–11.8] *p* < 0.0001) were associated with a higher one-year mortality rate.

**Table 6:** uni- and multivariate analysis of the risk factors of mortality at one year after LT

	Univariate analysis		Multivariate analysis	
Risk factors	Unadjusted Odds Ratio [95% CI]	*P value	Adjusted Odds Ratio [95% CI]	*P value
Pneumonia	5.9 [2.7–13.3]	< 0.0001	7.1 [2.7–18.7]	< 0.0001
BRI	0.5[0.2–1.6]	0.24	–	–
MRSA-MSSA BRI	0.9 [0.4–2.0]	1	–	–
NFGNB BRI	1.6 [0.8–3.1]	0.2	–	–
Age > 60 yo	1.8 [0.9–3.6]	0.14	3.0 [1.3–7.3]	0.013
Double LT	1.4 [0.7–2.8]	0.46	–	–
Idiopathic fibrosis	0.6 [0.3–1.2]	0.17	–	–
PGD	3.1 [1–9.8]	0.05	2.9 [0.7–12.6]	0.14
ECLS	0.7 [0.3–1.7]	0.5	–	–
MV > 14 days	6.6 [3.2–13.9]	< 0.0001	5.1 [2.2–11.8]	< 0.0001
SOFA > 5	1.2 [0.6–2.4]	0.6	–	–
SAPSII > 35	1.1 [0.6–2.1]	0.9	–	–
Apache 2 > 16	0.5 [0.3–1.0]	0.06	0.5 [0,2–1,3]	0.15

*p < 0.05 was considered statistically significant. BRI = bacterial respiratory infections; MSSA = methicillin-susceptible *Staphylococcus aureus;* MRSA = methicillin-resistant *Staphylococcus aureus;* NFGNB = non-fermenting Gram-negative bacilli; LT = lung transplantation; PGD = primary graft dysfunction; ECLS = extracorporeal lung support; MV = mechanical ventilation; SOFA = Sequential Organ Failure Assessment; SAPSII = Simplified Acute Physiology Score

## Discussion

This study, based on a large cohort of 175 LT patients, determined the epidemiology, prognosis and outcome after BRI in the early postoperative period following LT. Donor and recipient colonization played a limited role in the development of BRI. BRI resulted in pneumonia due to the same micro-organism in 17% of cases. This study also confirms the poor prognosis of pneumonia after LT.

### Bacterial species/colonization

*Enterobacteriaceae, Streptococcus spp.* and *Pseudomonas aeruginosa* were the predominant species isolated in our cohort, in line with other studies analyzing the postoperative period after LT [1, 5, 18].

Only 21% of recipients developed BRI with a micro-organism cultured from donor samples. The majority of these pathogens were Gram-positive bacteria (especially staphylococci and streptococci) and *Enterobacteriaceae*.

### *Definition of pneumonia and* tracheobronchitis

All patients with a diagnosis of BRI in our center received antibiotics adapted to the species cultured. Our antibiotic policy differs from that described in several other published studies, in which only patients with pneumonia were treated by antibiotics [4], resulting in a lower selection pressure, but an increased risk of undertreating true pneumonia, and increased mortality rates, as reported by Iregui et al. [18]. Percentage adequacy in our population, published in a previous paper, demonstrated a high rate of MDR bacteria in cases of late pneumonia [14].

It can be very difficult to distinguish between tracheobronchitis and pneumonia during the postoperative period following LT. In 2010, the International Society of Heart and Lung Transplantation Infectious Diseases Council working group defined proven pneumonia in cardiothoracic transplant recipients on the basis of clinical, radiologic and laboratory criteria. According to the authors, this definition applied not only to the early phase of LT, but also to the subsequent phase. However, during this crucial LT postoperative period, several criteria for the diagnosis of pneumonia may be difficult to assess.

We observed the presence of these criteria in both tracheobronchitis and pneumonia. Low rates of fever or hypothermia can be easily explained by administration of steroids and immunosuppressive agents. High rates of leukocytosis/leukopenia are frequently reported in the LT postoperative period and are not specific to sepsis. For example, ischemia-reperfusion is an important confounding factor, especially during the first five postoperative days [19, 20]. Similarly, the characteristics, changes and quality of sputum are difficult to assess during the LT postoperative period. Ischemia-reperfusion syndrome and fluid administration during and after surgery can make sputum characteristics difficult to interpret. However, purulent sputum appeared to be a relevant factor in our cohort of patients. Cough, dyspnea, tachypnea, or pleural rub, rales or bronchial breath sounds are also nonspecific signs and appear to be totally inappropriate and of limited value during the postoperative period, especially in ventilated patients. While the PaO_2_/FiO_2_ ratio appears to be a relevant parameter in ICU patients for the diagnosis of VAP, this criterion can be misleading after LT due to the presence of ischemia/reperfusion syndrome, primary graft dysfunction, and atelectasis or lung collapse. Finally, chest X-ray changes are challenging issues in these patients with ischemia/reperfusion, pulmonary edema, atelectasis and lung collapse, pleural effusion or acute rejection. X-ray signs of infiltration could be a very sensitive criterion for pneumonia in these patients, but are associated with poor specificity, as illustrated by the high rate of these signs in patients with tracheobronchitis.

### Links between BRI, tracheobronchitis, pneumonia and outcome

Riera et al. recently described an increased mortality rate in patients with postoperative pneumonia after LT [1]. ICU mortality increased to 40% and the in-hospital mortality rate was 50% in patients who developed postoperative pneumonia. This finding highlights the severity of postoperative pneumonia, even when appropriate antibiotics are rapidly prescribed. Although some studies have not found any link between pneumonia and mortality [21, 22], mortality rates remain high in this disease, which can be explained by the severe immunocompromised status of these patients, especially during this early phase of the immunosuppressive protocol, with potentially limited access of antibiotics to the lung during this crucial period.

In this context, we compiled all cases of pneumonia in our cohort according to the definition proposed by Riera et al. and identified 42 episodes of pneumonia in 34 patients. As described by Riera et al., the major finding is that, compared to tracheobronchitis, postoperative pneumonia is associated with dramatically increased 28-day (32%) and one-year (61%) mortality.

With a 28-day mortality of 7% and a one-year mortality of 19%, the outcome of patients with tracheobronchitis with no episode of pneumonia did not differ from that reported in other studies conducted during the LT postoperative period [1, 18]. However, no significant difference in terms of demographic and microbiologic data was observed between patients with one or more episodes of pneumonia and patients with tracheobronchitis but no episode of pneumonia. In view of the high mortality rate in this context, it is essential to rapidly distinguish between these two entities in order to focus on patients with pneumonia. Purulent sputum seems to be a very strong and useful clinical criterion to distinguish tracheobronchitis from pneumonia. However, it is a very subjective decision to note sputum as purulent. Further studies are required to characterize this group of patients with poor outcome.

Lastly, only a small proportion of patients (*n* = 14) did not develop any form of lung infection during the early postoperative period. No general characteristics were identified to differentiate these patients from those with BRI. However, the mortality rate of this small subset of patients (21% at 28 day and 42% at one year) remains high. This high 28-day mortality rate can be explained by non-infectious causes, such as primary graft dysfunction or surgical complications, while infectious diseases, especially late pneumonia, were mainly responsible for the high one-year mortality rate.

### Therapeutic consequences of our findings

The findings of this study cannot be used to determine whether our local policy to treat all episodes of tracheobronchitis was able to decrease the incidence of early-onset pneumonia in our population.

Most episodes of pneumonia were due to species unrelated to the donor’s or recipient’s colonization flora, as also observed in other studies in lung transplant cohorts. For example, in the study by Riera et al. analyzing 170 patients, most cases of pneumonia were totally independent of colonization prior to the graft [1]. Riera et al. observed only 3/20 cases of pneumonia caused by species cultured prior to the graft. In Riera’s study, gastroparesis was significantly associated with both pneumonia and ventilator-associated tracheobronchitis. Although most emerging organisms were not cultured from the host’s respiratory tract, we cannot exclude a host digestive tract origin of these bacteria. The gastric reservoir is a well-known source of contamination, which has led many teams to propose the use of selective decontamination.

### Limitations

Our study presents a number of limitations. Firstly, this single-center study reflects our local practice and essentially concerned COPD and idiopathic pulmonary fibrosis (IPF) patients. The epidemiologic findings would therefore probably be different in cystic fibrosis patients. Secondly, bacterial resistance was not evaluated in this study, but could be a relevant parameter, especially as all cases of BRI in our cohort were treated. We have previously reported a progressive increase of resistance rates, which could be related to our local antibiotic policy [14].

Thirdly, analysis of the inflammatory status was not included in our decision-making process, which is based exclusively on microbiologic data. The specific host inflammatory response could be an important trigger to stratify the development of tracheobronchitis or pneumonia. However, we have previously shown that the early inflammatory response is a confounding factor for the diagnosis of early pneumonia [12].

Fourthly, the pathogenic role of enterococci and coagulase-negative staphylococci (coNS) in pneumonia remains controversial, at least in immunocompetent ICU patients [23]. Enterococci and coNS BRI were treated according to our local rules. However, this policy is subject to debate and could be considered to be excessive.

Fifthly, respiratory colonization samples performed in a large proportion of patients more than 6 months before transplantation may have been a source of bias. Information about species during the last 6 months in our practice might be interesting, especially to determine the most appropriate antibiotic prophylaxis during the first 48 h following transplantation.

Lastly, this was a retrospective study and prospective evaluation of our practice could be a useful approach in future studies.

## Conclusion

In our cohort of immunocompromised patients after LT, 92% of patients developed BRI during the ICU postoperative period. Donor and recipient colonization played a limited role in the development of BRI. It can be difficult to distinguish between tracheobronchitis or pneumonia in the early postoperative phase after LT due to major confounding factors. Finally, our data confirm the poor prognosis of pneumonia after LT. Pneumonia has a major impact on mortality, which cannot be explained by microbiologic or demographic differences.

## Abbreviations

BA: bronchial aspiration
BRI: bacterial respiratory infections
CFU: colony-forming units
coNS: coagulase-negative staphylococci
ECLS: extracorporeal lung support
ICU: intensive care unit
LRTI: lower respiratory tract infections
LT: lung transplantation
MRSA: methicillin-resistant *Staphylococcus aureus*
MSSA: methicillin-susceptible *Staphylococcus aureus*
NFGNB: non-fermenting Gram-negative bacilli
PGD: primary graft dysfunction
VAP: ventilator-associated pneumonia
WBC: white blood cell
